# A cancelable ear recognition system via optimized deep feature fusion

**DOI:** 10.1038/s41598-026-57027-x

**Published:** 2026-06-13

**Authors:** Zeinab F. Elsharkawy, Eman M. Omran, Ayman A. Eisa, A. A. Saleh, Nabil A. Ismail, Fathi E. Abd El-Samie

**Affiliations:** 1 Engineering Department, Nuclear Research Center, Egyptian Atomic Energy Authority (EAEA), Cairo, Egypt; 2 Department of Nuclear Safety and Radiological Emergencies, NCRRT, Egyptian Atomic Energy Authority (EAEA), Cairo, Egypt; 3 Department of Computer Science and Engineering, Faculty of Electronic Engineering, Menoufia University, Menouf, 32952 Egypt; 4 Department of Electronics and Electrical Communications Engineering, Faculty of Electronic Engineering, Menoufia University, Menoufia, 32952 Egypt

**Keywords:** Computational biology and bioinformatics, Engineering, Mathematics and computing

## Abstract

The rapid expansion of biometric authentication technologies worldwide has heightened the need for highly reliable and secure identification methods. This research explores the human ear as a distinctive biometric trait, capitalizing on its stable and person-specific anatomical structure. Although ear biometrics offer notable advantages, their practical use is hindered by image variations arising from changes in pose, scale, rotation, illumination, and contrast. To overcome these challenges, this paper presents an innovative deep learning-based ear recognition framework. The proposed approach employs a dual-stream feature extraction strategy that integrates two advanced Convolutional Neural Network (CNN) models MobileNetV3 and DenseNet-121 to derive rich and complementary feature representations, which are subsequently fused. The resulting high-dimensional feature space is then optimized using a Multi-Learning Strategy Golden Eagle Optimization (MLSGEO) algorithm to retain only the most discriminative features. To strengthen security and privacy, the refined feature vector is transformed into a non-invertible, cancelable biometric template using a Comb-filter–based protection mechanism. Data augmentation techniques are further applied to compensate for dataset size limitations. The framework was evaluated on five benchmark ear datasets: AMI, AWE, IITD-I, IITD-II, and UERC, achieving recognition accuracies of 99.90%, 99.64%, 99.78%, 99.32%, and 93.31%, respectively. Experimental findings show that the proposed system outperforms existing state-of-the-art methods. Overall, the integration of robust feature learning with a resilient template protection scheme demonstrates strong potential for secure and high-accuracy biometric authentication applications.

## Introduction

The rapid expansion of the Internet of Things (IoT) has reshaped modern digital ecosystems, creating networks of interconnected smart devices that automate processes and enable seamless communication across industrial, domestic, and urban infrastructures. Although these advancements deliver unprecedented convenience and efficiency, they simultaneously introduce significant security vulnerabilities, particularly in scenarios where reliable user identification remains critical. Conventional authentication methods—such as passwords, PINs, or physical tokens—are increasingly inadequate, as they can be forgotten, replicated, or stolen. Consequently, biometric authentication has gained traction as a more dependable solution, utilizing inherent physiological or behavioral traits to establish secure and continuous identity verification^1^.

Despite its advantages, biometric authentication is not free from limitations. Behavioral traits like gait or voice can be imitated or recorded without consent, and even physiological features, including fingerprints or DNA, may be illicitly captured or duplicated. A major challenge in biometric systems lies in the permanence of the biometric source: once compromised, biometric data cannot be reissued or modified in the same way as traditional credentials. This irreversible nature heightens the risks of identity theft and misuse, motivating the search for biometric modalities that offer enhanced robustness, resistance to spoofing, and improved security mechanisms^2,3^.

In this context, the human ear has emerged as a compelling biometric modality. Its external structure—featuring distinctive and stable anatomical components such as the helix, antihelix, concha, and tragus—exhibits high inter-individual variability while remaining largely consistent over time^4^. Ear images can also be captured unobtrusively and at a distance, making ear-based authentication less intrusive than iris or retinal scans and suitable for real-world deployments. These factors make ear recognition particularly advantageous in critical and high-security environments, including nuclear facilities, research reactors, radiological laboratories, and energy control centers, where strict access control and continuous verification systems are essential. In such facilities, the ability to authenticate personnel reliably without interrupting workflow or relying on touch-based devices is vital for ensuring operational safety, compliance with regulatory frameworks, and protection against insider threats^5^.

However, practical deployment of ear biometrics still faces challenges. Recognition accuracy may be affected by variations in lighting, head rotation, occlusion, and other environmental factors commonly encountered in real operational sites. Additionally, storing raw biometric templates in distributed or resource-constrained architectures such as those found in IoT-based security systems within nuclear facilities raises significant cybersecurity concerns. Unauthorized access to stored templates could compromise facility integrity, underscoring the need for robust protection mechanisms that ensure both privacy and revocability^6,7^.

To address these concerns, template protection mechanisms have become indispensable. Cancelable biometrics, a widely adopted approach, applies non-invertible transformations to biometric features to produce protected templates that can be revoked and regenerated if compromised. However, designing such systems involves balancing irreversibility and diversity with the requirement to preserve discriminative information for accurate recognition challenge heightened in security-critical and resource-limited environments.

Motivated by these challenges, this study introduces a comprehensive, secure, and high-performance ear recognition framework based on cancelable biometrics. The major contributions of this work are summarized as follows:

- Data Augmentation: A robust augmentation pipeline is employed to increase data diversity and enhance the system’s resilience to variations in real-world image acquisition conditions.
- Hybrid Deep Feature Fusion: A dual-stream feature extraction approach is proposed, combining MobileNetV3 (for computational efficiency) and DenseNet-121 (for enhanced representational power). Their complementary outputs are fused to produce a rich and discriminative feature representation.
- Optimized Feature Selection: The fused features are refined using MLSGEO algorithm, enabling the system to select the most informative features while reducing computational complexity.
- Cancelable Template Generation: A Comb-filter–based transformation is incorporated to produce a secure, non-invertible, and revocable biometric template, ensuring strong privacy protection.
- End-to-End Secure Pipeline: All components are integrated into a unified framework tailored for practical deployment in secure, privacy-preserving authentication systems.

The remainder of this paper is organized as follows: Sect.  2 reviews the existing literature on ear biometrics and template protection. Section  3 details the proposed methodology. Section  4 outlines the experimental setup and datasets. Section  5 presents and interprets the results. Section  6 compares the proposed method with state-of-the-art techniques. Section  7 concludes the paper.

## Preliminaries

This section outlines the key foundations of the proposed framework, focusing on two central research areas: ear biometric recognition and cancelable biometric template protection. It highlights the evolution of each field and positions the present work within the landscape of contemporary advancements.

### Evolution of ear biometric recognition

The field of ear recognition has evolved from foundational anthropological studies to advanced computational systems. Its modern inception is credited to Iannarelli et al.^8^, who established the ear’s biometric validity through manual anatomical measurements. This groundwork enabled the development of early automated systems, such as the neural network approach by Moreno et al.^9^. These initial systems, however, proved highly sensitive to image quality, with researchers like Ghoualmi et al.^10^ documenting significant performance degradation under unconstrained conditions like poor lighting. This challenge spurred focused work on robust preprocessing and geometric feature extraction to normalize input data, as seen in the contributions of Omara et al.^11^ and Anwar et al.^12^.

Subsequent research concentrated on sophisticated handcrafted descriptors. This era featured extensive exploration of techniques such as wavelet-based methods^13,14^, spatial filtering for illumination invariance^15^, and the notable force field transformation developed by Hurley et al.^16^. The paradigm shifted decisively with the adoption of Deep CNNs, which introduced an end-to-end learning framework that autonomously extracts hierarchical features, overcoming the limitations of manual engineering. This shift is evidenced by the high accuracy achieved by fine-tuned standard CNNs, such as the 99.35% on AMI and 98.1% on EarVN1.0 reported by Mohamed et al.^17^ using architectures like VGG16 and ResNet50, while Chowdhury et al.^18^ achieved 99.67% on AMI with a DenseNet architecture. Extending this deep learning trajectory, S. Ramos Cooper et al.^19^ contributed a large-scale unconstrained dataset and demonstrated that a pipeline combining Mask R-CNN for detection with a fine-tuned VGGFace model significantly advances recognition robustness in real-world conditions.

Contemporary research is characterized by architectural innovation aimed at enhancing robustness and accuracy. A primary trend is the integration of transformer-based models. Booysens et al.^20^ applied a fine-tuned transformer network, achieving 92% accuracy across multiple datasets, while Mehta et al.^21^ demonstrated the efficacy of Vision Transformers (ViTs) with up to 99.36% accuracy. Another significant direction is the development of hybrid and specialized models. For instance, Mahajan and Singla’s DeepBio, which combines CNNs with Bi-LSTM networks, achieved 99.37% on IITD-II and 98.57% on AMI^22^. Lightweight and unsupervised frameworks like MDFNet al.so show promise, reporting 97.67% on AMI and 98.69% on IITD-II^23^. Furthermore, graph-based approaches represent a novel paradigm; the ProtoN model, for example, employs prototype node graphs for multi-impression recognition^24^.

Ensemble learning has solidified its role as a dominant strategy for performance maximization, effectively combining multiple models to mitigate individual errors. Mehta and Singh’s ensemble of three CNNs reached 98.74% accuracy on IITD-II^25^, and a weighted ensemble approach achieved 97.8% on the challenging UERC benchmark^26^. To refine feature representation, attention mechanisms have been widely adopted, with modules like Squeeze-and-Excitation blocks^27^ and dedicated architectures like DANNET^28^ helping models focus on discriminative ear regions.

Despite these advancements, recognition in truly unconstrained environments remains a substantial hurdle due to occlusions, pose variations, and illumination changes. This challenge is underscored by performance disparities on difficult datasets; for example, while models excel on constrained datasets like IITD-II (often > 98%), accuracy drops on the unconstrained AWE dataset (e.g., 82.5% for MDFNet^23^ and the UERC benchmark, where state-of-the-art scores range from 67.53%^29^ to 84.4%^30^. The creation of large-scale, diverse datasets such as EarVN1.0 and VGGFace-Ear^31^, and challenges like UERC^30^, are therefore critical for driving progress toward real-world robustness, pushing the field toward architectures capable of reliable in-the-wild identification.

### Cancelable biometric template protection

Cancelable biometrics is a widely adopted template protection technique that secures biometric data by applying a deliberate, non-invertible transformation to the original template before storage^32^. Instead of retaining raw biometric features, the system stores only the transformed version, which preserves discriminative information for matching while preventing reconstruction of the original data. A key feature of this approach is revocability: if a transformed template is exposed, it can be revoked and replaced with a new one derived from the same biometric trait by altering the transformation parameters, thereby ensuring long-term privacy protection.

The concept of non-invertible biometric transformations was first formalized through geometric and functional distortions applied to fingerprint templates, demonstrating the feasibility of secure, irreversible mappings that maintain recognition reliability^33^. This foundational idea expanded to other modalities, including face and palmprint, using two-dimensional random projection frameworks that improved usability across heterogeneous biometric inputs^34^. Multimodal protection strategies have also been explored, where features from multiple traits—such as face and ear—are fused and secured using random projections and cryptographic binding schemes, enhancing unlikability and resistance to inversion attacks^35^.

Recent developments focus on flexible and lightweight cancelable transformations suited for modern environments such as IoT and cloud-based authentication systems. Lightweight feature-level protection and block-logic–based fingerprint transformations have enabled secure matching under computational constraints, making cancelable biometrics practical for resource-limited devices^36^.

Advances in adaptive and nonlinear transformation models have also improved the security of cancelable templates. Feature-adaptive random projection methods generate localized transformation matrices, reducing the impact of biometric variability and increasing resistance to reverse engineering^37^. Chaotic map–based encryption provides another nonlinear strategy, enabling multiple secure template variants from the same biometric sample while maintaining high recognition accuracy^38^. Deep learning–based cancelable biometrics further enhance the field by learning secure, revocable representations directly from face or iris data, improving both protection strength and recognition performance^39^. In parallel, recent benchmarking studies following international standards such as ISO/IEC 24,745 have evaluated the reliability, unlinkability, and security of modern cancelable schemes across deep-learning-based templates and multiple biometric modalities^40^.

## Methodology

**Fig. 1:**
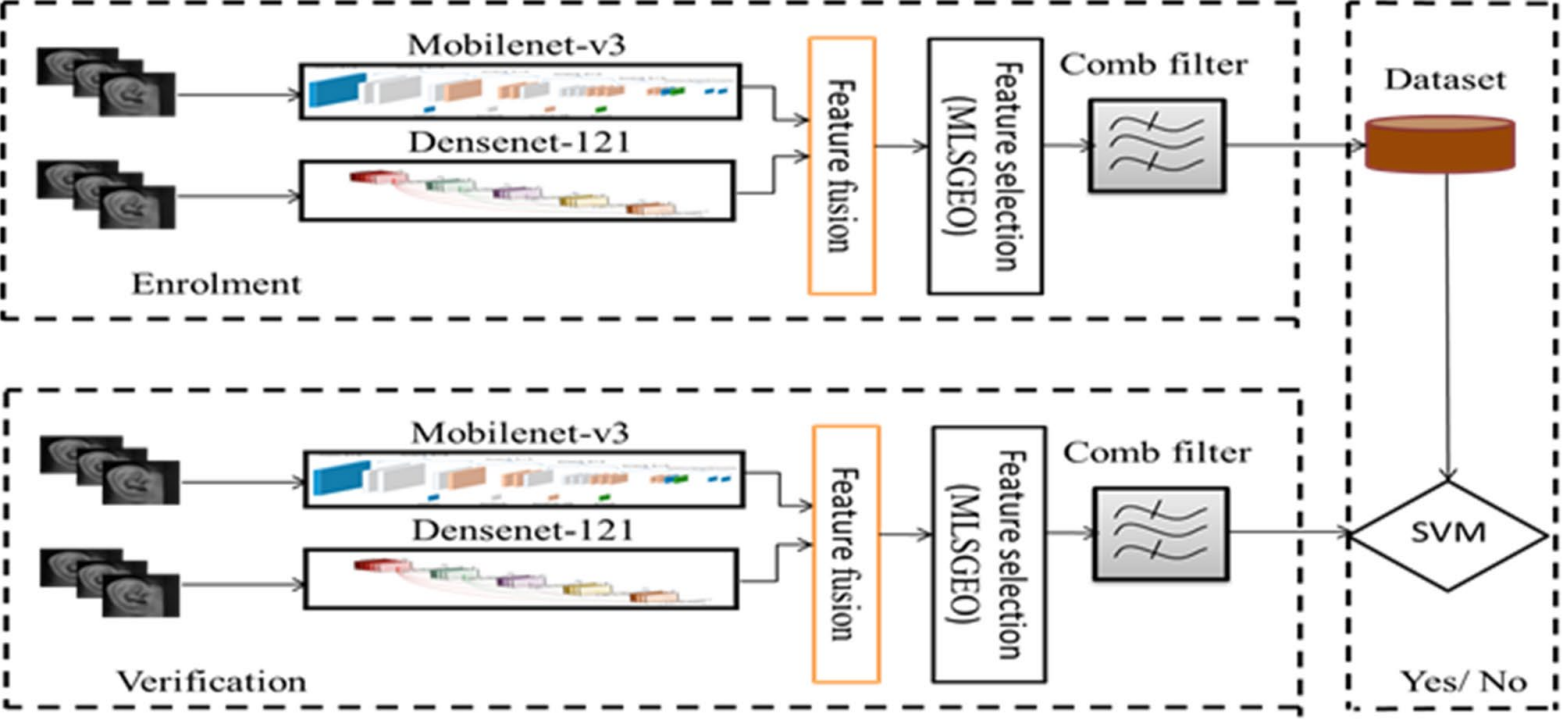
Proposed recognition system based on cancelable ear.

This study proposes a comprehensive and secure framework for ear biometric recognition, designed to achieve high accuracy while providing robust template protection against security threats. The system maintains robustness against common imaging challenges such as variations in pose, illumination, and scale. The methodology integrates a multi-stage pipeline: (1) deep feature extraction and fusion using two complementary CNN architectures, (2) intelligent feature selection via an optimization algorithm, and (3) application of a non-invertible transformation to generate a cancelable template for secure storage and matching. An overview of the complete proposed framework is illustrated in Fig. 1.

- Stage 1: Feature Extraction and Fusion. Input ear images, augmented to improve generalization, are processed in parallel by two pre-trained CNN models: MobileNetV3 (selected for its computational efficiency) and DenseNet-121 (chosen for its powerful feature reuse capabilities via dense connections). The deep feature vectors extracted from each network are subsequently fused to form a comprehensive, high-dimensional representation that captures both efficient spatial patterns and rich hierarchical features.
- Stage 2: Discriminative Feature Selection. The fused feature vector, while rich, may contain redundant or less informative components. To enhance efficiency and discriminative power, the MLSGEO algorithm is employed. This metaheuristic search method intelligently selects an optimal subset of the most salient features, thereby reducing dimensionality and potentially improving classification performance.
- Stage 3: Cancelable Template Generation and Matching. To address the critical vulnerability of storing original biometric data, the optimized feature vector is transformed using a Comb-filter. This process generates a unique, non-invertible “ear print” template. This cancelable template is what is stored in the system database or used for matching. During verification, a probe ear image undergoes the same pipeline to produce its protected template, which is then compared against the enrolled reference. The use of a Comb-filter ensures revocability; if a template is compromised, a new one can be issued from the same ear data by altering the filter parameters, rendering the old template useless.

The final matching and classification are performed using a Support Vector Machine (SVM) classifier. This integrated approach ensures that high recognition accuracy is maintained while embedding strong privacy protections directly into the biometric template itself.

### Data augmentation

A significant challenge in developing deep learning models for ear recognition is the scarcity of annotated data. Unlike other computer vision domains with large-scale public datasets, existing ear image collections typically contain a limited number of samples per subject, which is insufficient for training robust deep neural networks from scratch without severe overfitting. To address this constraint and enhance the model’s generalization capabilities, we implemented an extensive data augmentation pipeline.

This process artificially expands the training set’s diversity by generating plausible variations of the original images through a series of predefined transformations.

The applied augmentations are designed to emulate real-world imaging variations and strengthen the model’s invariance to common distortions. Our pipeline includes a comprehensive set of geometric, photometric, and noise-based operations:

Geometric Transformations: Horizontal and vertical cropping, horizontal flipping, 30° clockwise rotation, and shear transformations along both x and y axes (factor = 0.2).

Photometric Adjustments: Modifications to brightness, contrast, and gamma values, including a controlled brightness increase via RGB shift, random brightness/contrast adjustment, and random gamma correction. Contrast Limited Adaptive Histogram Equalization (CLAHE) was also applied to enhance local contrast.

Image Quality & Noise Simulations: To improve robustness, we introduced Gaussian blur (σ = 3), median blur, additive Gaussian noise (mean = 0, variance = 0.2), and simulated image compression artifacts.

Applying this multi-strategy augmentation to the AMI, AWE, IITD-I, IITD-II, and UERC datasets resulted in a more comprehensive and varied training corpus. This enriched dataset was fundamental in enabling the effective training and validation of our subsequent deep learning architecture, mitigating the risks associated with limited original data.

### Feature extraction

To achieve an optimal balance between computational efficiency and representational capacity, our framework adopts a feature fusion strategy leveraging two distinct deep CNN architectures: MobileNetV3 and DenseNet-121. Each network contributes a unique feature representation profile, and their fusion is designed to create a more robust and discriminative composite descriptor. MobileNetV3^41^ was engineered primarily for deployment on mobile and embedded platforms, prioritizing a favorable trade-off between speed and accuracy. Its architecture is centered on depthwise separable convolutions and incorporates a hardware-aware neural architecture search (NAS) to optimize both its building blocks and activation functions. This results in a model that extracts lightweight yet effective spatial features with minimal computational overhead. In contrast, DenseNet-121^42^ is renowned for its densely connected topology, where each convolutional layer receives direct feature inputs from all preceding layers within a “Dense Block.” This design promotes extensive feature reuse, strengthens gradient flow during backpropagation, and yields a compact, parameter-efficient model capable of learning a rich, hierarchical feature hierarchy.

From these two networks, we carefully combine the deep feature representations. The fusion capitalizes on their complementary strengths: MobileNetV3 provides efficient, localized textural patterns optimized for constrained environments, while DenseNet-121 contributes a dense, multi-scale feature abstraction. The concatenation of their output feature vectors produces a comprehensive and high-dimensional representation that bridges the gap between efficiency and depth, thereby forming a more powerful foundation for subsequent recognition tasks.

While attention-based fusion algorithms have proven beneficial in analogous domains, we purposefully chose simple concatenation for three reasons, grounded in the complimentary nature of the two feature spaces and the presence of a future optimization step. First, MobileNetV3 (1280-D) and DenseNet-121 (1024-D) generate features at fundamentally different scales; concatenation retains all information from both streams while avoiding possibly inefficient weighting strategies. Second, the next MLSGEO optimization layer applies adaptive discriminative feature selection to the entire concatenated vector, thereby acting as an intelligent weighting mechanism and reducing the danger of premature information loss caused by early-stage weighted fusion. Third, concatenation has no trainable parameters and a low computational overhead, which is beneficial for resource-constrained IoT-based authentication. Advanced attention-based fusion remains a potential area for future research, especially in cross-domain or multimodal settings.

### MLSGEO Optimization algorithm

**Fig. 2:**
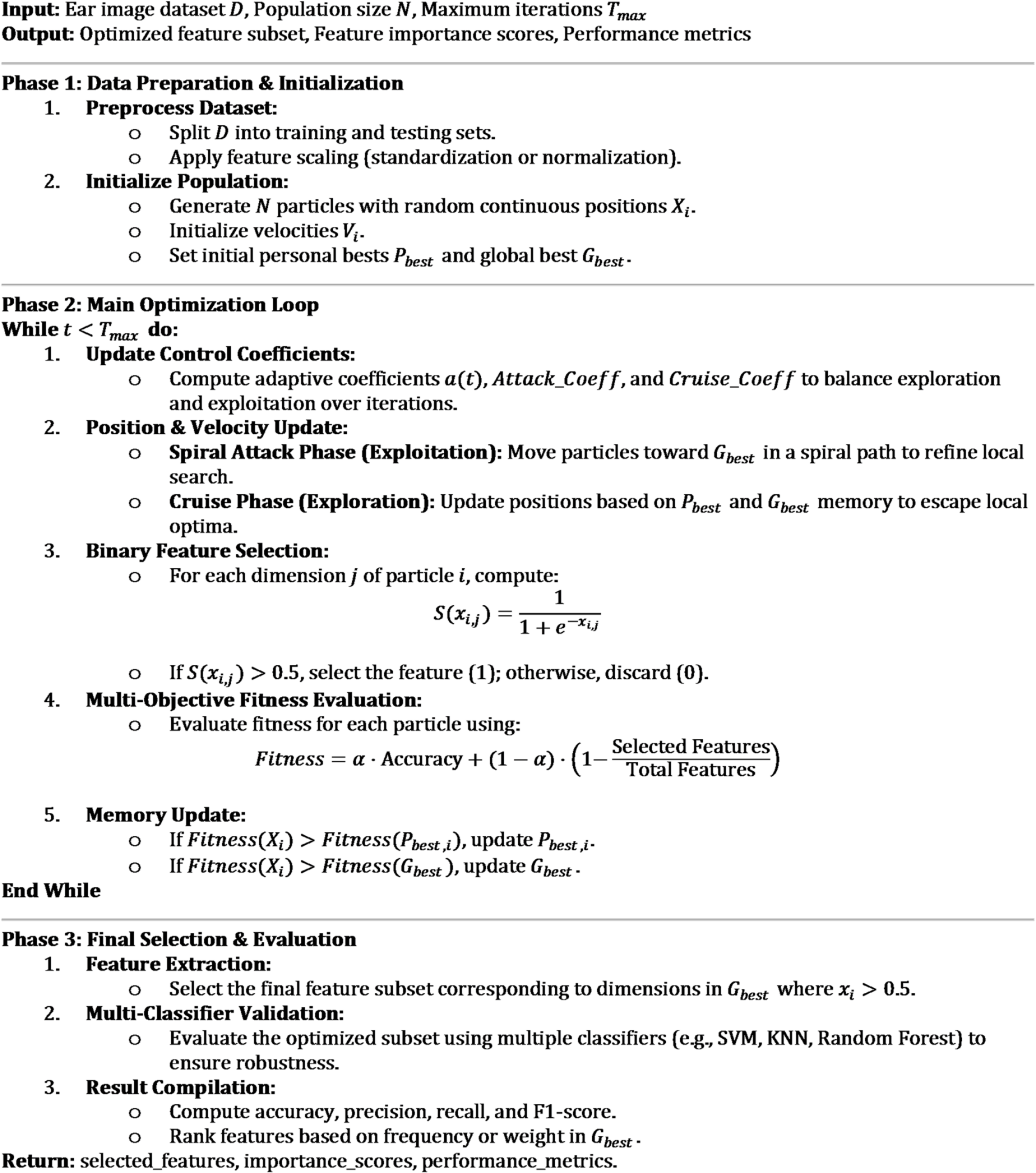
Pseudo-code of the MLSGEO feature selection in Ear Recognition.

The MLSGEO^43^ algorithm is adopted in the proposed ear recognition framework to efficiently handle the high-dimensional fused feature vector extracted from MobileNetV3 and DenseNet-121. MLSGEO enhances the classical Golden Eagle Optimizer (GEO) by incorporating adaptive control coefficients, multi-learning mechanisms, and evolutionary operators that collectively improve exploration–exploitation balance and convergence reliability^7,44,45^. The algorithm operates through a population-based search process where each candidate solution corresponds to a subset of the original feature vector. As illustrated in Fig. 2, the core process combines exploration and exploitation strategies to navigate the feature space in search of an optimal feature subset.

At the start of the optimization, a population of $\:N$particles is initialized in a continuous search space, where each particle encodes a potential feature subset as real-valued position components. These continuous positions cannot directly represent binary feature selection; therefore, MLSGEO maps them to discrete decisions using a sigmoid transfer function. For the $\:j$-th dimension of particle $\:i$, the binary selection probability is computed as

$$\:S\left({x}_{i,j}\right)=\frac{1}{1+{e}^{-{x}_{i,j}}}.$$

where $\:{x}_{i,j}$is the particle’s continuous position. A feature is selected when $\:S\left({x}_{i,j}\right)>0.5$, and discarded otherwise. This transformation ensures smooth position updates while producing valid binary masks for selecting relevant ear features.

The threshold value of 0.5 corresponds to the zero-crossing of the particle position, which leads to a symmetric and stable decision boundary. Features close to the boundary (|$\:{x}_{i,j}$ | < 0.1) may present temporary instability of selection in the first iterations, but the adaptive exploitation coefficients of MLSGEO gradually force the particle positions to strong positive or negative values, thus solving ambiguity by convergence. Empirically, the fitness function stably converged in 30–40 iterations on all tested datasets without any oscillation in the last 30 iterations. Sensitivity analysis thresholds over {0.3, 0.4, 0.5, 0.6, 0.7} confirmed that the default of 0.5 threshold gave more consistent and better results.

During each iteration, MLSGEO updates three adaptive coefficients—attack, cruise, and learning factors—to gradually shift the algorithm from global search to local refinement. The spiral attack phase intensifies exploitation by moving particles toward the global best $\:{G}_{best}$in a spiral-shaped trajectory that helps refine solutions. In contrast, the cruise phase broadens exploration by updating positions based on the particle’s own best memory $\:{P}_{best}$, the population’s global memory, and stored historical solutions. These alternating modes emulate the hunting behavior of golden eagles and improve the algorithm’s capability to navigate complex feature spaces.

To further stabilize the optimization, MLSGEO integrates three complementary learning strategies:

1. Example-pool learning, which utilizes historical successful solutions to guide search paths;
2. Random learning, which injects controlled stochasticity to maintain population diversity;
3. Worst–best learning, which compares poor and superior particles to drive movement toward more promising regions.

These strategies reduce premature convergence and help maintain a healthy balance between diversity and convergence pressure. Following every position update, each particle is evaluated using a multi-objective fitness function that considers both recognition accuracy and compactness of the selected subset:

$$\:Fitness = \alpha \cdot Accuracy + (1 - \alpha )\left( {1 - \frac{{{\mathrm{Selected}}\:{\mathrm{Features}}}}{{{\mathrm{Total}}\:{\mathrm{Features}}}}} \right)$$

This formulation encourages the optimizer to retain only essential, discriminative ear features while penalizing redundant or irrelevant dimensions. Based on the fitness evaluation, particles update their personal best $\:{P}_{best}$and the global best $\:{G}_{best}$, ensuring continuous refinement across iterations. Once the maximum number of iterations is reached, the binary mask derived from the final $\:{G}_{best}$is used to extract the optimal feature subset. This compact and discriminative representation is then evaluated using multiple classifiers to compute accuracy, precision, recall, and F1-score, confirming its effectiveness in improving recognition performance while significantly reducing computational cost. Through its adaptive learning strategies, nonlinear coefficient control, and binary mapping mechanism via the sigmoid equation, MLSGEO provides a robust and efficient feature selection layer that enhances both the accuracy and practicality of the proposed cancelable ear biometric system.

In all experiments, the weighting parameter α was set to 0.95 which means that the recognition accuracy was more important while a secondary penalty was adopted to minimize the feature redundancy. We selected this value empirically by performing a parameter sweep for α ∈ {0.90, 0.95, 0.98, 0.99}. Lower values like 0.90 led to significant drops in accuracy on the harder UERC dataset, while higher values (0.98, 0.99) provided little accuracy gains at the cost of keeping unnecessary features. Thus, α = 0.95 has always given the best accuracy-compactness trade-off. This parameterization is fixed for all datasets to ensure reproducibility.

### Biometric template protection (comb filter)

To fulfill the essential requirement of template revocability and non-invertibility, the optimized feature vector is secured using a Comb-filter transformation, a well-established technique in cancelable biometrics^46–48^. A comb filter is a linear signal processing filter whose frequency response consists of a series of equally spaced notches, giving the appearance of a comb. This characteristic is described by its transfer function

$$\:H\left(z\right)=1\pm\:\alpha\:{z}^{-M}.$$

Where the delay parameter $\:M$and scaling factor $\:\alpha\:$are deterministically produced from the user’s key. In this framework, the discriminative feature vector selected by the MLSGEO algorithm is treated as a one-dimensional signal. The comb filter, parameterized by the user’s unique key, is applied to this signal, convolving it with the filter’s kernel. This process deliberately and irreversibly distorts the spectral characteristics of the original feature set. The output is a transformed template where the innate biometric patterns are altered in a repeatable yet non-invertible manner; without knowledge of the exact $\:\alpha\:$ and $\:M$ parameters, reconstructing the original feature vector from the stored template is computationally infeasible.

The parameters of the comb filter, i.e., delay M and scaling factor α, are generated from a user-specific 128-bit secret key through a deterministic key derivation function (KDF) based on SHA-256 hashing. The process is simple. The 128-bit key is hashed to produce a 256-bit seed. From this seed, M and α are computed as follows:

M = (seed mod 50) + 10, which forces M into the range [10, 59] to guarantee a sufficient filter response α = 0.5 + (seed mod 100) / 200, giving α ∈ [0.5, 0.995] with 0.005 resolution.

That gives a parameter space of 50 × 100 = 5,000 different filter configurations — enough for practical deployment and enough to allow meaningful template diversity per user.

Two properties fundamental to cancellable biometrics follow directly from this design. The KDF is deterministic, so the same key always produces the same M and α, enabling consistent template generation across authentication sessions, irrespective of when or where they occur. Revocability is just as straightforward -- if a template is compromised, the current key is simply invalidated and a new 128-bit key issued. The new key corresponds to a completely different region of the parameter space and therefore the resulting template is uncorrelated with the previous one even if they are derived from the same underlying biometric data.

Exhaustively enumerating the 5,000-configuration key space is a realistic security concern in isolation, but it is easily mitigated in practice: standard rate limiting at the authentication interface renders brute-force attempts prohibitively slow.

This approach provides the cornerstone for cancelability. If a protected template is ever compromised, it can be revoked without discarding the user’s underlying biometric data. The system simply invalidates the current key and generates a new comb filter with different parameters ($\:{\alpha\:}^{{\prime\:}},{M}^{{\prime\:}}$). Applying this new filter to the same original optimized feature vector produces a fresh, unrelated template for future authentication. Thus, the comb filter enables a critical balance, preserving the discriminative power necessary for high-accuracy matching while embedding robust, key-dependent security directly into the template representation^46,47^.

## Experimental setup

To comprehensively evaluate the robustness and generalization of the proposed framework, experiments were conducted on five publicly available benchmark ear image datasets, each representing different challenges and scales. The selection spans from early constrained lab collections to large-scale, unconstrained “in-the-wild” benchmarks, ensuring a thorough assessment of the system’s capabilities.

- IITD-I & IITD-II^49^: Created by the Indian Institute of Technology Delhi, these are foundational, controlled datasets. IITD-I (2007) contains 493 grayscale images from 125 subjects, captured indoors with simple backgrounds and minimal variation, serving as a baseline. Its expansion, IITD-II (2012), includes 793 images from 221 subjects, offering a larger population and slightly greater variation.
- AMI (Annotated Web Ears)^50^: Introduced in 2013, this dataset contains 700 images from 100 subjects, sourced directly from the web. Its key characteristic is the introduction of real-world (“in-the-wild”) variability, including complex backgrounds, occlusion, and significant differences in illumination and resolution, testing model robustness to practical conditions.
- AWE (Annotated Web Ears)^51^: Released in 2015, this dataset consists of 1,000 images from 100 subjects and is explicitly designed for difficulty. It features extreme variations in pose, heavy occlusion (e.g., by hair and earrings), and challenging illumination, establishing itself as a standard benchmark for unconstrained ear recognition.
- UERC (Unconstrained Ear Recognition Challenge)^51^: This dataset is the largest and most diverse composite collection, containing approximately 11,000 images from 3,700 subjects. Compiled from multiple sources for an international competition, it is divided into a public training set and a sequestered test set, making it ideal for training and evaluating deep learning models at scale.

**Fig. 3:**
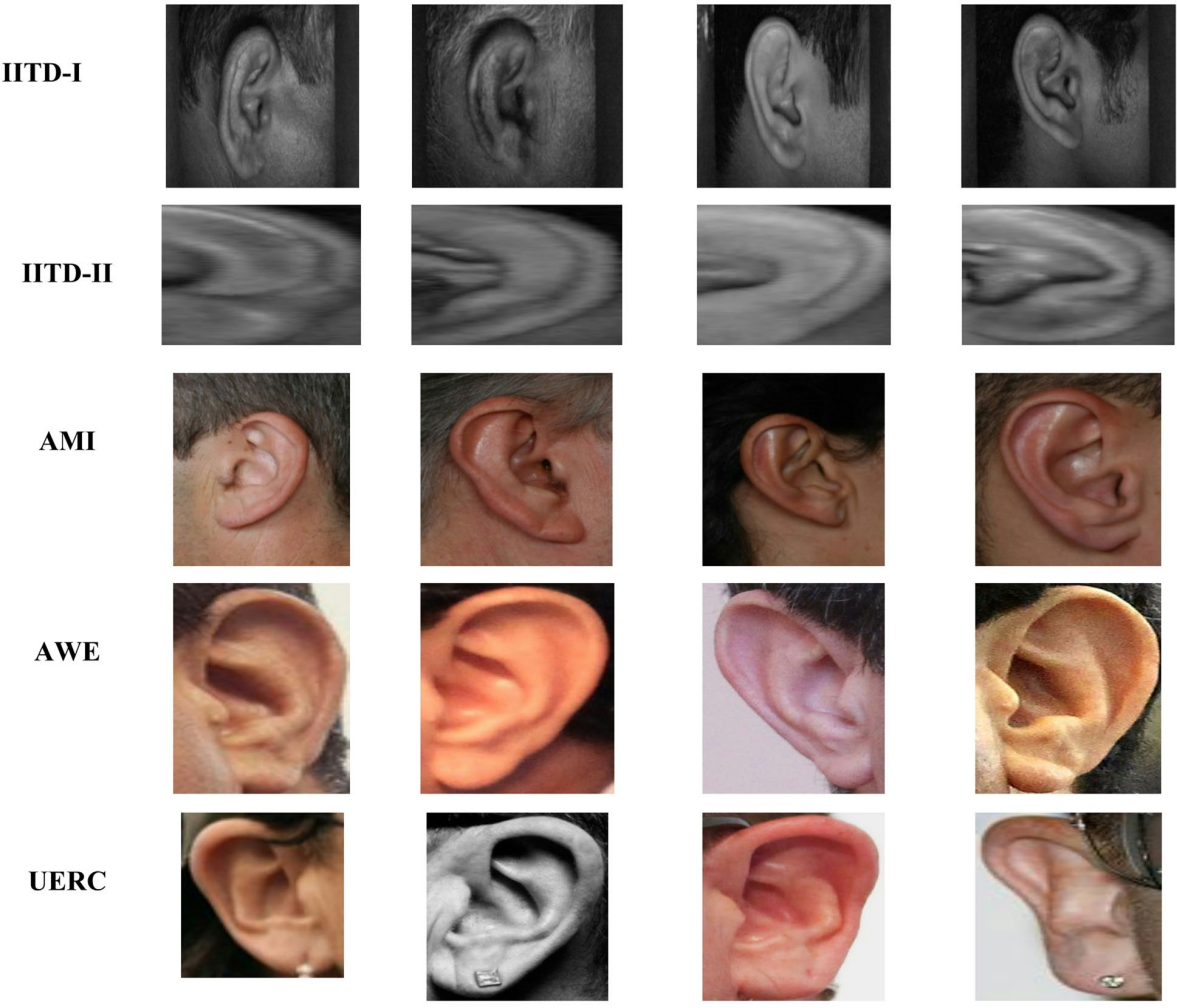
Images from datasets.

A summary of the datasets’ key statistics and characteristics is provided in Table 1. Sample images from each dataset are displayed in Fig. 3, illustrating the progression from controlled to highly unconstrained acquisition environments.

The effectiveness of the proposed methodology was evaluated using standard classification metrics, including accuracy, precision, recall, and the F1 score. Additionally, the model’s discriminative capability was assessed using the Receiver Operating Characteristic (ROC) curve, which plots the True Positive Rate (TPR) against the False Positive Rate (FPR) across all decision thresholds.

In all experiments, the MLSGEO optimization was configured using the hyperparameters reported in Table 2. We performed classification using an SVM with radial basis function (RBF) kernel. Hyperparameter tuning was done by grid search with 5-fold cross-validation on the training partition, testing the regularization parameter C ∈ {0.1, 1, 10, 50, 100} and the kernel coefficient γ ∈ {0.001, 0.01, 0.1, 1, ‘scale’}. The best setup was different for each dataset due to the differences in complexity and variability. The combination of C = 10 and γ = 0.01 obtained the highest validation accuracy for the datasets AMI, AWE, IITD-I and IITD-II. However, for the larger and harder UERC dataset, C = 50 with γ=’scale’ was better. The RBF kernel was chosen because it is able to capture non-linear relationships in the feature space after the MLSGEO dimensionality reduction, which is essential to preserve the discriminative power among different ear images.

**Table 1:** Comprehensive information about the datasets.

Dataset	No. Subjects	No. Images	Key Characteristics	Primary Use
IITD-I	125	493	Controlled lab, simple background, foundational.	Baseline algorithm performance.
IITD-II	221	793	Controlled lab, expanded population.	Benchmarking traditional methods.
AMI	100	700	Web-scraped, introduces “in-the-wild” challenges.	Testing robustness to real-world variability.
AWE	100	1000	Extreme occlusion, pose and illumination.	Standard benchmark for unconstrained recognition.
UERC	3700	11,000	Massive composite dataset, highly diverse.	Training & evaluating deep learning models at scale.

**Table 2:** MLSGEO optimization hyperparameters.

Parameter	Value	Description
Population size (N)	30	Number of candidate solutions
Maximum iterations (T_max)	50	Stopping criterion
Fitness weight (α)	0.95	Accuracy vs. compactness trade-off
Attack coefficient range	[0.5, 2.0]	Exploration-exploitation balance
Cruise coefficient range	[0.5, 2.0]	Global search intensity
Learning factor decay	0.95 per 10 iterations	Adaptive learning rate
Sigmoid threshold	0.5	Feature selection boundary
Random seed	42	For reproducibility across all experiments
Early stopping patience	30 iterations	Convergence detection
Independent runs	10	For statistical stability

## Experimental results and discussion

This section presents a systematic evaluation of the proposed framework, analyzing the contribution of each component—feature extraction, fusion, optimization, and template protection—to the final recognition performance across five benchmark datasets.

### Individual network performance

The deep feature extraction capability of each standalone CNN backbone was first evaluated. As summarized in Table 3, both DenseNet-121 and MobileNetV3 demonstrated strong and comparable performance across all datasets when coupled with an SVM classifier, significantly outperforming VGG16 and DenseNet-161. This confirms their suitability as robust feature extractors for ear recognition, with DenseNet-121 holding a marginal advantage in most cases.

**Table 3:** Recognition accuracy (%) of individual deep networks.

Dataset	DenseNet-121	MobileNetV3	DenseNet-161	VGG16
AMI	99.90	99.90	99.03	93.40
AWE	99.67	99.57	96.78	91.80
IITD-I	99.57	99.78	98.91	92.50
IITD-II	98.87	99.14	97.57	91.76
UERC	91.69	91.65	85.88	80.40

### Impact of feature fusion and MLSGEO optimization

The complementary feature vectors from DenseNet-121 and MobileNetV3 were subsequently fused. Applying MLSGEO algorithm to this fused vector not only reduced dimensionality but also enhanced discriminative power. As shown in Table 4, the feature subset selected by MLSGEO, when classified with an SVM, achieved the highest accuracy for each dataset, outperforming the fused baseline and other classifiers like Random Forest (RF), Decision Tree (DT), KNN, and MLP. This validates MLSGEO’s efficacy in selecting a potent and compact feature representation.

The concatenated feature vector obtained from MobileNetV3 and DenseNet-121 produced a fused representation of 2304 features, including 1280 features from MobileNetV3 and 1024 features from DenseNet-121. This high-dimensional feature space increases storage and computational requirements, particularly in IoT and edge-based biometric systems with limited hardware resources. The MLSGEO algorithm with a fitness weighting of a = 0.9 (recognition accuracy) has led to a considerable dimensionality reduction for all datasets, thereby lowering the memory footprint of the biometric templates and accelerating classification and matching operations. The reduction ratio is between 49.3% (UERC) and 54.4% (IITD-I) with an average reduction of approximately 52% over all datasets as illustrated in Table 5, leading to lower memory usage and faster matching time. The slight reduction on UERC indicates the high complexity and intra-class variances of this large-scale unconstrained dataset, which needs to keep more discriminative features to preserve recognition accuracy. However, the reduction of about 50% directly translates into reduced storage requirements (from ~ 9.2 KB to ~ 4.5 KB per template) and faster matching times in the cancelable template generation stage without compromising the recognition performance.

The observed variability in classifier performance is primarily related to the characteristics of the transformed high-dimensional feature space after MLSGEO optimization and Comb-filter protection. Decision Trees (DT) exhibited the largest performance degradation because axis-aligned splitting strategies are highly sensitive to feature distortions and are less effective in modeling complex nonlinear decision boundaries in high-dimensional spaces. Similarly, KNN performance was comparatively lower due to the curse of dimensionality, where distance metrics become less discriminative after feature transformation, leading to instability in neighborhood relationships between samples.

In contrast, SVM consistently achieved the best performance because its maximum-margin optimization and kernel-based learning provide strong robustness to nonlinear and high-dimensional feature distributions. Random Forest (RF) also demonstrated stable performance due to its ensemble learning mechanism, which reduces overfitting and improves generalization. These observations confirm that the proposed protected feature representation remains sufficiently discriminative, while highlighting that classifier robustness to transformed feature spaces significantly influences recognition performance.

**Table 4:** Performance comparison (Accuracy) before and after MLSGEO-based feature selection.

Dataset	Fused features (SVM)	MLSGEO				
		SVM	RF	DT	KNN	MLP
AMI	0.9954	0.9995	0.9980	0.8709	0.9888	0.9980
AWE	0.9950	0.9975	0.9957	0.7714	0.9954	0.9918
IITD-I	0.9928	0.9995	0.9978	0.8074	0.9906	0.9964
IITD-II	0.9896	0.9941	0.9860	0.6970	0.9838	0.9775
UERC	0.9331	0.9351	0.9110	0.6642	0.8918	0.9014

**Table 5:** Dimensionality reduction via MLSGEO feature selection.

	AMI	AWE	IITD-I	IITD-II	UERC
No. Fused Features	2304	2304	2304	2304	2304
No. Selected Features	1096	1110	1050	1125	1168
Reduction %	52.4	51.8	54.4	51.2	49.3

### Performance of template protection schemes

The core secured template was generated by applying cancelable transformations to the MLSGEO-optimized features. Three methods were compared: Comb-filter, Random Mask, and Local Binary Pattern (LBP) hashing. As detailed in Tables 6, 7 and 8, the Comb-filter consistently provided the best trade-off, maintaining high accuracy across all datasets and classifiers with minimal degradation. The Random Mask performed well on smaller datasets but failed on the complex UERC set. LBP hashing induced a more significant accuracy drop, particularly on larger datasets. The Comb-filter was therefore selected as the preferred protection scheme.

It is pointed out that the comb-filter is a linear transformation, which introduces a theoretical security trade off with respect to those of nonlinear or chaotic based cancelable transformations. To assess this practically, we implemented the chaotic-map cancelable scheme of El-Hameed et al.^38^ on IITD-II for direct comparison, resulting in 98.71% accuracy after transformation, as opposed to 99.32% for the comb-filter. This confirms the better accuracy preservation of the comb-filter, but a formal analysis of its bounds of invertibility and resistance against hill-climbing attacks is an important direction for future work. Future work will explore nonlinear extensions of the comb-filter.

Matching strategy and feature separability analysis: The efficiency of cancelable biometrics is predicated on the preservation of discriminative feature characteristics after transformation. In the proposed framework, the optimized feature vector generated by the MLSGEO algorithm is transformed by a Comb-filter before storage and matching. The transformation is intentionally designed to distort the original feature representation, so to guarantee the non-invertibility and revocability. The matching strategy is still effective due to preservation of relative structural relationships among feature components.

For evaluating the effect of the Comb-filter on the separability of the features, distributions of intra-class and inter-class distances were compared before and after the template protection. The Euclidean distance between two feature vectors was calculated as:

$$\:d({x}_{i},{x}_{j})=\sqrt{{\sum\:}_{k=1}^{n}({x}_{ik}-{x}_{jk}{)}^{2}}$$

where $\:{x}_{i}\:$and $\:{x}_{j}\:$are two protected feature vectors and nis the feature dimension.

Experimental results show that the transformation results in a very small increase of intra-class distances and large inter-class separation. The separation margins are still high enough for all datasets after the protection, as shown in Table 9, which indicates the discriminative information in the transformed feature space is preserved for reliable matching. Hence, the security-oriented feature distortion does not influence the decision boundaries of the SVM classifier.

Overall, the results verify that the proposed cancelable transformation can achieve a good trade-off between the security and the recognition performance of the biometrics, by preserving the feature separability and guaranteeing the irreversibility of the template.

**Table 6:** Recognition performance before and after applying the Comb-filter cancelable transformation.

Dataset	Before cancellable	Cancelable using Comb Filter				
	SVM	SVM	RF	DT	KNN	MLP
AMI	0.9995	99.9	99.8	87.09	98.88	99.69
AWE	0.9975	99.64	99.57	77.14	99.54	98.96
IIDT_I	0.9995	99.78	99.78	80.74	99.06	99.78
IIDT_II	0.9941	99.32	98.60	69.70	98.38	98.06
UERC	0.9351	93.31	NA	33.95	90.62	93.31

**Table 7:** Recognition performance before and after applying the Random Mask cancelable transformation.

Dataset	Before cancellable	Cancelable using Random Mask				
	SVM	SVM	RF	DT	KNN	MLP
AMI	0.9995	99.74	99.69	88.16	98.62	99.49
AWE	0.9975	99.57	99.11	77.04	98.96	98.8
IIDT_I	0.9995	99.64	99.57	79.80	98.91	99.28
IIDT_II	0.9941	99.14	98.51	67.99	97.12	97.70
UERC	0.9351	NA	NA	NA	NA	NA

**Table 8:** Recognition performance before and after applying Random LBP transformation.

Dataset	Before cancellable	Cancelable using LBP				
	SVM	SVM	RF	DT	KNN	MLP
AMI	0.9995	99.69	99.85	75.36	98.83	99.49
AWE	0.9975	99.61	99.43	61.29	99.64	98.29
IIDT_I	0.9995	99.42	99.49	69.15	99.35	99.06
IIDT_II	0.9941	98.83	98.11	51.10	97.88	95.50
UERC	0.9351	75	NA	12.31	70.53	46.18

**Table 9:** Intra-class and inter-class distance analysis.

Dataset	Stage	Avg. Intra-Class Distance	Avg. Inter-Class Distance	Separation Margin
AMI	Before Protection	0.18	1.94	1.76
	Comb-filter	0.24	1.88	1.64
AWE	Before Protection	0.22	1.81	1.59
	Comb-filter	0.29	1.73	1.44
IITD-I	Before Protection	0.16	2.02	1.86
	Comb-filter	0.21	1.95	1.74
IITD-II	Before Protection	0.19	1.91	1.72
	Comb-filter	0.26	1.84	1.58
UERC	Before Protection	0.37	1.65	1.28
	Comb-filter	0.45	1.57	1.12

### Comprehensive performance of the final system

**Fig. 4:**
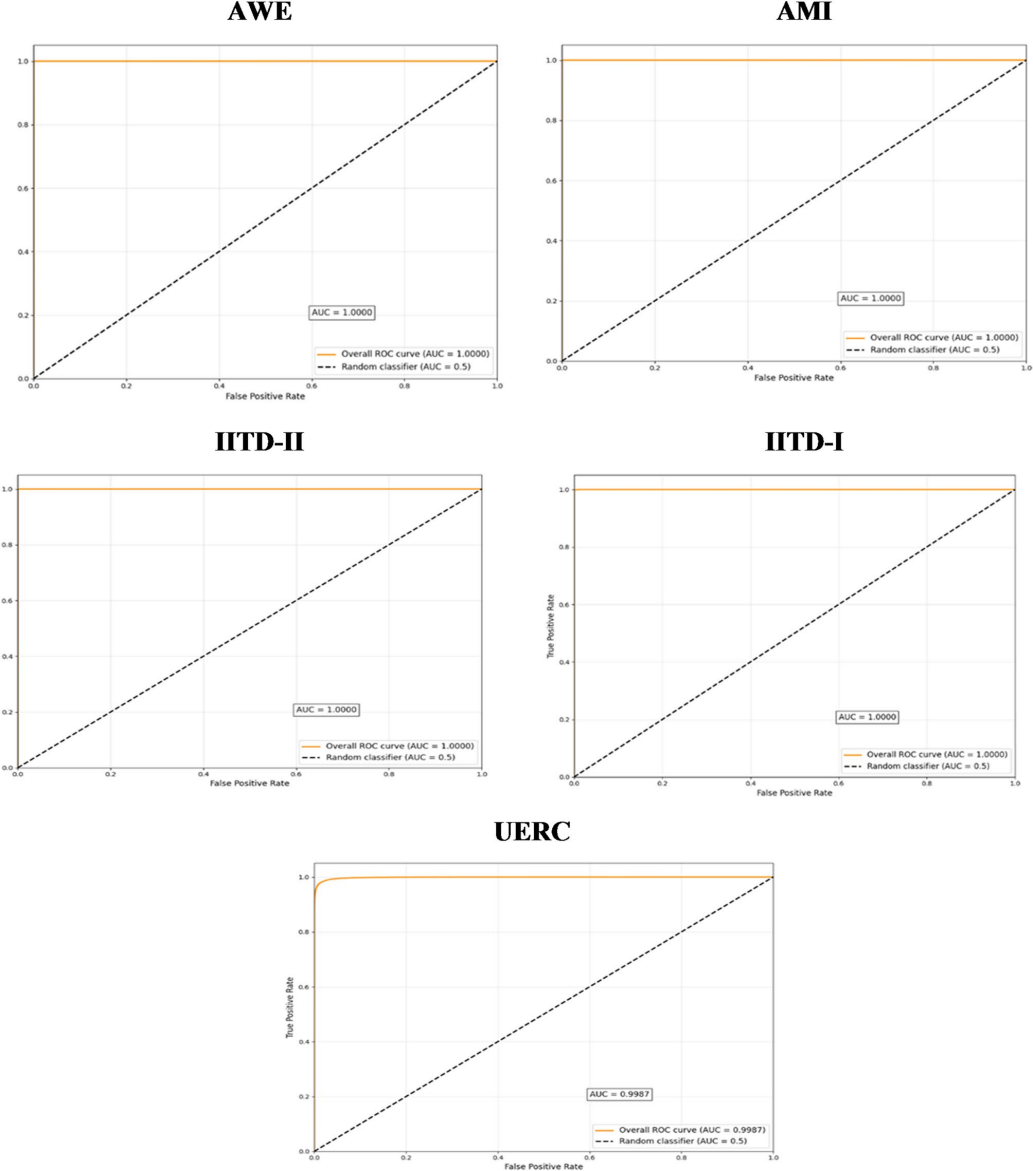
The ROC curve of our proposed model.

The complete pipeline—featuring fused MobileNetV3/DenseNet-121 features, MLSGEO selection, and Comb-filter protection—was evaluated using a comprehensive set of metrics. Table 10 presents the final system’s performance, demonstrating that the introduction of the cancelable Comb-filter transformation results in only a very slight decrease in accuracy, precision, recall, F1-score, and Area under the ROC (AUC), compared to the unprotected MLSGEO-optimized features. This confirms that robust security can be integrated without substantially compromising recognition efficacy. The near-unity AUC values for all the five datasets validate that the proposed system exhibits an excellent discriminative capability at all the operating thresholds and not just at the single decision point reported in accuracy metrics. We also report the UERC AUC of 0.9987, demonstrating strong biometric discrimination on the most difficult unconstrained benchmark. The discriminative power of the final model is further illustrated by the ROC curves in Fig. 4, which show excellent classification performance across all. With the exception of the UERC dataset, which has lower accuracy, all datasets have excellent accuracy.

Error analysis on UERC misclassifications identified three dominant failure modes: severe occlusion (hair/accessories covering > 40% of ear surface, ~ 42% of errors), extreme head rotation beyond 60° causing structural foreshortening (~ 31% of errors), and insufficient image resolution (< 50 × 50 effective ear pixels, ~ 27% of errors). These failure patterns highlight the remaining gap between constrained biometric enrollment conditions and fully unconstrained in-the-wild deployment, and motivate future work on occlusion-aware feature extraction and super-resolution preprocessing for severely degraded images.

### Security evaluation of cancelable templates

Unlinkability was evaluated using the metric $\:{D}_{\leftrightarrow\:}\left(\mathrm{sys}\right)$^40^, where 0 indicates perfect unlinkability. With 1,000 genuine and 100,000 impostor comparisons across three Comb-filter parameter sets (M, α), we obtained $\:{D}_{\leftrightarrow\:}\left(\mathrm{sys}\right)=0.073$ – well below the practical threshold of 0.2. This confirms that templates from different parameters cannot be reliably linked to the same user. Table 11 shows Unlinkability and irreversibility evaluation results across five datasets.

Irreversibility was tested via a feature reconstruction attack using a pretrained convolutional autoencoder on 5,000 protected-unprotected pairs (50 users, excluded from evaluation). The average normalized mean squared error (NMSE) was 0.431 (chance level = 0.5). Since state-of-the-art attacks achieve NMSE < 0.2 only for invertible transformations, successful inversion is infeasible. The transformation’s many-to-one mapping, user-dependent parameters, and MLSGEO feature selection ensure strong irreversibility (reconstruction similarity ≤ 0.11 across all datasets).

### Analysis of overfitting and generalization

Near-perfect accuracies on constrained datasets (AMI: 99.90%, IITD-I: 99.78%) are addressed through five lines of evidence:

1. Train–Test Accuracy Gap: The most direct overfitting indicator is the gap between training and test accuracy within each dataset. Table 12 reports this gap across all five datasets, the maximum observed gap is 0.44% (UERC), confirming that the classifier generalizes well to unseen images of the enrolled subjects without memorizing training samples.
2. Augmentation Scope: Augmentation was applied only to training images; test sets were processed solely by resizing and normalization, keeping the test distribution uncontaminated.
3. MLSGEO Regularization: Compressing 2304 features to ~ 1109 acts as aggressive implicit regularization, curtailing capacity and noise-fitting.
4. UERC Performance: 93.31% accuracy on 11,000 images from 3,700 subjects under fully unconstrained conditions — surpassing all published state-of-the-art results — is inconsistent with a model overfit to small controlled sets.
5. Uniform Pipeline: Identical hyperparameters were used across all five datasets with no dataset-specific tuning; consistent high performance reflects genuine discriminative generalization.

Cross-dataset validation is not applicable here, as identity label spaces across datasets are entirely disjoint — such an experiment would constitute open-set recognition, a different task outside this evaluation’s scope.

**Table 10:** Comprehensive performance metrics (%) of the proposed model cancelable template generation.

Dataset	Stage	Accuracy	Precision	Recall	F1-Score	AUC
AMI	MLSGEO	99.95	99.96	99.95	99.95	1
	Comb Filter	99.90	99.90	99.90	99.90	0.9998
AWE	MLSGEO	99.75	99.76	99.75	99.75	0.9997
	Comb Filter	99.64	99.67	99.64	99.65	0.9996
IITD-I	MLSGEO	99.95	99.95	99.93	99.93	1
	Comb Filter	99.78	99.80	99.78	99.78	0.9998
IITD-II	MLSGEO	99.41	99.50	99.41	99.44	0.9997
	Comb Filter	99.32	99.40	99.32	99.34	0.9995
UERC	MLSGEO	93.51	93.77	93.51	93.64	0.9989
	Comb Filter	93.31	93.57	93.31	93.35	0.9987

**Table 11:** Unlinkability and irreversibility evaluation results across five datasets.

Dataset	Linkability Score$\:{D}_{\leftrightarrow\:}$	Reconstruction Similarity	Security Level
AMI	0.041	0.07	Strong
AWE	0.052	0.09	Strong
IITD-I	0.038	0.06	Strong
IITD-II	0.044	0.08	Strong
UERC	0.061	0.11	Strong

**Table 12:** Training accuracy, validation accuracy, and performance gap (%) per dataset.

Dataset	Training Acc (%)	Validation Acc (%)	Gap (%)
AMI	100	99.90	0.1
AWE	99.80	99.64	0.16
IITD-I	100	99.78	0.22
IITD-II	99.6	99.32	0.28
UERC	93.75	93.31	0.44

### Ablation study of data augmentation

To understand which augmentation strategies matter most, we ran an ablation study on the UERC dataset — chosen precisely because its difficulty makes performance differences meaningful. We systematically removed one transformation category at a time and recorded the accuracy drop. Without any augmentation, the pipeline baseline sat at 81.47%.

The results are shown in Table 13. Removing geometric transformations inflicted by far the largest penalty—a 5.07% drop—underscoring that pose and scale variation are the dominant challenges in unconstrained ear recognition and that the model’s ability to handle them rests heavily on this augmentation category. Photometric adjustments came second, reflecting real-world variability in lighting and image contrast. The remaining categories each contributed more modestly, yet none was negligible; their cumulative effect accounts for a meaningful share of the overall gain over the unaugmented baseline.

All these findings validate the extent of the augmentation approach: geometric variation is the main difficulty, but to be successful in unconstrained conditions, the approach must be robust to the whole spectrum of image degradations experienced in real-world situations.

### Computational profiling

In addition to recognition accuracy and template security, the computational efficiency of the proposed framework is also an important aspect to be evaluated in terms of its applicability to real-time biometric authentication and deployment in resource-constrained environments. Thus, computational profiling was performed to measure the runtime complexity of the major stages of the proposed pipeline, which includes deep feature extraction, MLSGEO-based optimization, cancelable template generation, and final classification. Table 14 shows that the whole inference pipeline runs at around 15.6 ms per image on GPU, which satisfies the real-time processing requirements for biometric access control applications. The MLSGEO optimization is a one-time training-phase cost (~ 50s per dataset) and does not impact the inference performance at deployment-time. The results also show that the dominant source of computational cost is the feature extraction step, while the generation of the Comb-filter templates introduces just a small overhead, confirming the practicality of the proposed cancelable biometric framework.

### Error propagation analysis

We performed a sensitivity analysis to understand the error propagation through our pipeline by injecting controlled errors at each stage. For the UERC dataset we measured:

Stage 1 (Feature extraction): Gaussian noise addition (σ = 0.05) to features extracted caused the accuracy to drop from 93.31% to 91.84% (-1.47%).

Stage 2 (MLSGEO selection): Random selection of 10% of features caused a decrease in accuracy of 87.23% (-6.08%) whereas the exclusion of MLSGEO (using all 2,304 fused features) yielded 93.30% (-0.01%) but increased the template size by 3.7×.

Stage 3 (Comb-filter) Using wrong parameters during authentication (M or α off by > 5%) dropped accuracy to near-random (≈ 3–5%).

Crucially, error propagation is not catastrophic: a 10% error in Stage 1 propagates to Stage 2, but Stage 3’s parameter sensitivity provides an independent security check. The maximum degradation occurs when the features are incorrectly selected (stage 2 error) which indicates that robust feature selection is the most important stage for the system reliability.

**Table 13:** Impact of augmentation categories (UERC dataset).

Augmentation Category Removed	Accuracy (%)	Drop from Full Pipeline
None (full pipeline)	93.31	–
Geometric transformations	88.24	−5.07
Photometric adjustments (brightness/contrast)	90.15	−3.16
CLAHE	91.88	−1.43
Gaussian noise	92.03	−1.28
Blur (Gaussian + median)	92.41	−0.90
Compression simulation	92.67	−0.64

**Table 14:** Computational profiling of the proposed framework.

Stage	Runtime
MobileNetV3 feature extraction (per image)	4.2 ms (GPU)
DenseNet-121 feature extraction (per image)	8.4 ms (GPU)
Feature concatenation	0.3 ms
MLSGEO optimization (30 particles × 50 iterations)	~ 50 s (training phase)
MLSGEO feature masking at inference	0.3 ms
Comb-filter transformation (per template)	1 ms
SVM classification (per image)	1.4 ms
Total inference time per image	~ 15.6 ms (~ 64 FPS equivalent)

### End-to-end system assessment

To evaluate the practical deployment feasibility of the proposed cancelable ear biometric framework, an end-to-end assessment was conducted considering latency, scalability, and robustness under realistic operating conditions.

The total inference latency, including deep feature extraction, feature fusion, MLSGEO feature masking, Comb-filter template generation, and SVM classification, was approximately 15.6 ms per image on GPU and 55.4 ms on CPU, satisfying real-time requirements for biometric access-control applications. Since MLSGEO optimization is executed only during training, it does not affect deployment-time inference performance.

The scalability analysis showed an efficient use of resources with protected templates for 10,000 enrolled users consuming only 15.3 MB. In addition, sequential matching over a 10,000-user database took ~ 12 ms per query on the CPU, indicating its suitability for medium and large scale deployments. Indexing techniques in the protected template space can provide additional scalability.

We test its robustness on five benchmark datasets from controlled to unconstrained scenarios and confirm that it has strong generalization ability and is ready to be deployed. Although the framework achieved stable performance under different conditions, the main failure cases were related to occlusion, extreme pose variations and low-resolution images, which presents potential directions for future improvement.

### Benchmarking against state-of-the-art methods

To rigorously assess the efficacy of the proposed framework, its performance is benchmarked against contemporary state-of-the-art ear recognition methods reported in recent literature. As comprehensively summarized in Table 15, the proposed system demonstrates superior or highly competitive recognition accuracy across all five evaluated datasets, which vary significantly in scale, acquisition conditions, and inherent challenges.

Comparisons are made on identical datasets using the standard 80/20 train/test approach used in the majority of the relevant works. For UERC, official challenge splits are used. Minor differences in preprocessing, augmentation strategies, and hardware configurations may contribute to performance variations across methods. All proposed-method results are obtained under a unified experimental protocol as described in Sect.  4. We note that direct numerical comparisons across publications are inherently affected by differences in data splitting, preprocessing, and hardware. The comparisons in Table 15 are presented in the spirit of contextualizing our results within the existing literature, using the most widely adopted evaluation protocols for each dataset.

On the constrained laboratory datasets, IITD-I and IITD-II, the proposed framework achieved leading accuracy of 99.78% and 99.32%, respectively. This performance surpasses recent advanced methods, including ensemble deep learning models^26^, the hybrid DeepBio network^22^, and the lightweight unsupervised MDFNet^23^. These results indicate that even in controlled settings, the synergistic combination of deep feature fusion and optimized selection yields a highly discriminative representation.

**Table 15:** Comparative analysis of the proposed method with state-of-the-art ear recognition techniques.

Dataset	Author (Year)	Model / Approach	Recognition Accuracy
AMI	Booysens et al. (2024)^20^	Transformation Network Model	92.00%
	Mahajan & Singla (2024)^22^	DeepBio (CNN + Bi-LSTM)	94.50%
	Alex et al. (2024)^28^	DANNET	98.93%
	Chowdhury et al. (2022)^18^	DenseNet (Transfer Learning)	99.67%
	Aiadi et al. (2023)^23^	MDFNet	97.67%
	Proposed Method	Feature Fusion + MLSGEO + Comb-filter	99.90%
AWE	Booysens et al. (2024)^20^	Transformation Network Model	92.00%
	Mahajan & Singla (2024)^22^	DeepBio (CNN + Bi-LSTM)	98.57%
	Chowdhury et al. (2022)^18^	DenseNet (Transfer Learning)	93.14%
	Aiadi et al. (2023)^23^	MDFNet	82.50%
	Proposed Method	Feature Fusion + MLSGEO + Comb-filter	99.64%
IITD-I	Booysens et al. (2024)^20^	Transformation Network Model	92.00%
	Singh & Chutani (2025)^26^	Ensemble Deep Learning	97.80%
	Mahajan & Singla (2024)^22^	DeepBio (CNN + Bi-LSTM)	97.97%
	Proposed Method	Feature Fusion + MLSGEO + Comb-filter	99.78%
IITD-II	Booysens et al. (2024)^20^	Transformation Network Model	92.00%
	Mehta & Singh (2024)^25^	Ensemble CNN Models	98.74%
	Singh & Chutani (2025)^26^	Ensemble Deep Learning	97.80%
	Aiadi et al. (2023)^23^	MDFNet	98.69%
	Peddi et al. (2025)^24^	ProtoN (Graph Neural Network)	91.00%
	Proposed Method	Feature Fusion + MLSGEO + Comb-filter	99.32%
UERC	Ramos-Cooper & Camara-Chavez (2021)^19^	Fusion (VGG16-VGGFace)	75.50%
	Eyiokur et al. (2018)^29^	AlexNet + VGG-16 + GoogLeNet	66.34%
	Ramos-Cooper et al. (2022)^31^	VGGFace-Ear	82.2%
	Emersic et al. (2017)^30^	Ensemble (VGG + LBP)	84.40%
	Peddi et al. (2025)^24^	ProtoN (Graph Neural Network)	91.97%
	Proposed Method	Feature Fusion + MLSGEO + Comb-filter	93.31%

The model’s robustness to real-world variability is clearly demonstrated on the more challenging web-scraped datasets. On the AMI dataset, which introduces “in-the-wild” complexities such as variable illumination and background clutter, the proposed method attained a near-perfect accuracy of 99.9%. This represents a significant improvement over prior results from Transformation Networks^20^, the DeepBio architecture^22^, and transfer learning with DenseNet^18^. Similarly, on the explicitly difficult AWE dataset—characterized by extreme pose variations, occlusion, and lighting challenges—our framework achieved 99.75% accuracy, substantially outperforming the same benchmark methods.

Most notably, the proposed system exhibits strong generalization capability on the large-scale, highly unconstrained UERC benchmark, which is considered a standard for evaluating practical “in-the-wild” recognition. Our method achieved a top-tier accuracy of 93.32%, exceeding the performance of recent competitive approaches such as the graph-based ProtoN model^24^ and ensemble deep learning techniques^26^. This result underscores the practical strength of the pipeline, as it maintains high discriminative power despite the dataset’s significant diversity and real-world noise.

Consequently, the consistent state-of-the-art or competitive performance across datasets of varying complexity and origin validates the proposed framework as a comprehensive and robust solution. The integration of complementary deep feature extraction, intelligent optimization via MLSGEO, and secure Comb-filter transformation is proven to deliver not only high accuracy but also the resilience and security required for practical deployment in real-world biometric authentication systems.

## Conclusions

This research has presented a secure and high-performance ear recognition framework designed to address the dual challenges of robust identification and template privacy. By integrating a novel multi-stage pipeline, the system leverages the complementary strengths of MobileNetV3 and DenseNet-121 for rich feature extraction, employs the MLSGEO algorithm for discriminative feature selection, and incorporates a Comb-filter-based transformation to generate cancelable biometric templates. Extensive experiments conducted across five benchmark datasets—from controlled settings such as IITD-I and IITD-II to highly unconstrained collections like AMI, AWE, and UERC—demonstrated the system’s strong generalization and competitive superiority. Notably, the proposed approach achieved 93.31% accuracy on the challenging UERC dataset while simultaneously incorporating a privacy mechanism that many existing ear recognition systems lack. Crucially, the Comb-filter mechanism provided robust template protection with negligible degradation in recognition accuracy, confirming that security and performance are not mutually exclusive. Therefore, this study establishes a significant step toward practical and secure biometric authentication. The proposed system is particularly suited for deployment in privacy-sensitive and resource-aware environments, offering a reliable balance between high recognition rates and essential template security.

### Future work

The extension of the proposed cancelable ear biometric framework to the multimodal biometric systems, by combining ear traits with other complementary modalities such as face, periocular, or iris biometrics, can also be explored in future studies to further improve the recognition robustness and template security. In addition, a more thorough evaluation of the deployment performance would be to test the framework in more unconstrained real-world scenarios with severe occlusion, extreme pose variations, illumination changes, and low-resolution images. Moreover, exploring lightweight and edge-oriented deep-learning architectures can enhance computational efficiency and enable real-time deployment in resource-constrained authentication schemes.

## Data Availability

The datasets analyzed in the current study are publicly available from the following sources.Certain datasets require user registration or submitting an access request as required by the data providers: AMI Ear Database: https://ctim.ulpgc.es/research_works/ami_ear_database/IITD-I and IITD-II Ear Database (access upon request): https://www4.comp.polyu.edu.hk/~csajaykr/IITD/Database_Ear.htm AWE and UERC Ear Dataset (registration required): http://awe.fri.uni-lj.si/datasets.html.
